# *Ginkgo biloba* Extract Individually Inhibits JNK Activation and Induces c-Jun Degradation in Human Chondrocytes: Potential Therapeutics for Osteoarthritis

**DOI:** 10.1371/journal.pone.0082033

**Published:** 2013-12-11

**Authors:** Ling-Jun Ho, Li-Feng Hung, Feng-Cheng Liu, Tsung-Yun Hou, Leou-Chyr Lin, Chuan-Yueh Huang, Jenn-Haung Lai

**Affiliations:** 1 Institute of Cellular and System Medicine, National Health Research Institute, Zhunan, Taiwan, R.O.C.; 2 Graduate Institute of Basic Medical Science, PhD Program of Aging, China Medical University, Taichung, Taiwan, R.O.C.; 3 Rheumatology/Immunology and Allergy, Department of Medicine, Tri-Service General Hospital, National Defense Medical Center, Taipei, Taiwan, R.O.C.; 4 Department of Orthopaedics, Tri-Service General Hospital, National Defense Medical Center, Taipei, Taiwan, R.O.C.; 5 Graduate Institute of Medical Science, National Defense Medical Center, Taipei, Taiwan, R.O.C.; 6 Division of Allergy, Immunology and Rheumatology, Department of Internal Medicine, Chang Gung Memorial Hospital, Chang Gung University, Tao-Yuan, Taiwan, R.O.C.; Northwestern University Feinberg School of Medicine, United States of America

## Abstract

Osteoarthritis (OA) is a common joint disorder with varying degrees of inflammation. The ideal anti-OA drug should have immunomodulatory effects while at the same time having limited or no toxicity. We examined the anti-inflammatory effects of *Ginkgo biloba* extract (EGb) in interleukin-1 (IL-1)-stimulated human chondrocytes. Chondrocytes were prepared from cartilage specimens taken from patients with osteoarthritis who had received total hip or total knee replacement. The concentrations of chemokines and the degree of cell migration were determined by ELISA and chemotaxis assays, respectively. The activation of inducible nitric oxide synthase (iNOS), mitogen-activated protein kinases (MAPKs), activator protein-1 (AP-1), and nuclear factor-kappaB (NF-κB) was determined by immunoblotting, immunohistochemistry, and electrophoretic mobility shift assay. We found that EGb inhibited IL-1-induced production of chemokines, which in turn resulted in attenuation of THP-1 cell migration toward EGb-treated cell culture medium. EGb also suppressed IL-1-stimulated iNOS expression and release of nitric oxide (NO). The EGb-mediated suppression of the iNOS-NO pathway correlated with the attenuation of activator protein-1 (AP-1) but not nuclear factor-kappaB (NF-κB) DNA-binding activity. Of the mitogen-activated protein kinases (MAPKs), EGb inhibited only c-Jun N-terminal kinase (JNK). Unexpectedly, EGb selectively caused degradation of c-Jun protein. Further investigation revealed that EGb-mediated c-Jun degradation was preceded by ubiquitination of c-Jun and could be prevented by the proteosome inhibitor MG-132. The results imply that EGb protects against chondrocyte degeneration by inhibiting JNK activation and inducing ubiquitination-dependent c-Jun degradation. Although additional research is needed, our results suggest that EGb is a potential therapeutic agent for the treatment of OA.

## Introduction

Osteoarthritis (OA) is a common joint disorder among people of advanced age. In addition to subchondral bone and synovial membrane, cartilage is recognized as one of the main targeted structures responsible for joint diseases in OA patients. Cartilage damage results from the failure of chondrocytes to maintain a homeostatic balance between matrix synthesis and degradation. In this regard, inflammation plays a pivotal role in cartilage damage and the pathogenesis of OA [1]. It has been demonstrated that plasma proteins present in OA synovial fluid can induce the production of inflammatory cytokines [2]. Interleukin (IL)-1, a well-known cytokine that plays a critical role in the immunopathogenesis of OA, is responsible for damaging cartilage by inducing matrix metalloproteinases (MMPs) and proteases [3]. Chondrocytes in OA patients have increased levels of expression of IL-1 receptors and are more susceptible to IL-1 stimulation than other cell types [4], and studies have shown that inhibition of IL-1 actions by an IL-1 receptor antagonist is beneficial in reducing the symptoms of OA. Furthermore, blocking IL-1-induced MMP gene expression by physiologic and pharmacologic inhibitors has been reported to be an important therapeutic approach for OA patients [1].

Chemokines are a group of inflammatory mediators that recruit leukocytes into inflamed joints and chemokine receptors are highly expressed on chondrocytes [5]. Chemokines can induce MMP production in chondrocytes and have been shown to contribute to the development of synovial inflammation in patients undergoing arthroscopic meniscectomy due to traumatic meniscal injury [6], [7]. Osteoarthritic chondrocytes are capable of producing chemokines such as RANTES (regulated upon activation, normal T cell expressed and secreted), macrophage inflammatory protein-1alpha (MIP-1α) and MIP-1β [5]. Furthermore, RANTES stimulates inducible nitric oxide synthase (iNOS) expression in OA chondrocytes as effectively as IL-1, resulting in cartilage degradation [8]. The release of NO leads to the amplification of inflammation and subsequent tissue injury [9]. Experimental OA models indicate that the inhibition of chemokines and NO production significantly reduces progression of cartilage damage [8]–[10].

Activator protein-1 (AP-1), a heterodimeric transcription factor comprising proteins belonging to the c-Jun and c-Fos families, plays important roles in many inflammatory processes and autoimmune diseases [11]. Both c-Jun and c-Fos proteins dimerize with many other basic leucine zipper proteins, expanding the number of potential AP-1-associated factors that bind to AP-1 sites [12]. This mechanism accounts for the cooperative regulation of the promoter regions in many cytokine and chemokine genes. AP-1 also regulates IL-1-induced transcriptional activation of MMP and iNOS genes [13], [14].

*Ginkgo biloba* extract (EGb) is widely sold as a phytomedicine in Europe and as a dietary supplement in the United States. EGb has been reported to be beneficial in the treatment of Alzheimer's disease, failing memory, dementia, cognitive impairment associated with premenstrual syndrome, cerebrovascular dysfunction and peripheral vascular disorders, and some other age-related disorders [15]–[18]. In addition to reducing proliferation of vascular smooth muscle cells, EGb has been shown to improve glucose homeostasis and reduce plasma high sensitivity C-reactive protein concentrations [17]. EGb has also been reported to be a useful adjuvant for the treatment of glaucoma [19]. Importantly, EGb does not affect the *in vivo* activity of the major cytochrome P450 enzymes in humans [20], [21]. We previously observed that EGb inhibits the activation of human peripheral blood T lymphocytes by suppressing the c-Jun N-terminal kinase (JNK)-AP-1 signaling pathway [22]. In addition, in a double-blind, placebo-controlled clinical trial we found that EGb resulted in improvement in joint pain among patients with OA (manuscript in preparation). Therefore, in the current study we investigated whether EGb has a protective effect in proinflammatory cytokine-stimulated human chondrocytes *in vitro*. We found that EGb elicits an immunomodulatory effect by inhibiting IL-1-induced JNK activation and degrading c-Jun protein in a ubiquitination-dependent manner in human chondrocytes.

## Materials and Methods

### Reagents and antibodies

Recombinant human IL-1β was purchased from R&D Systems, Inc. (St. Paul, MN). EGb powder, a standardized Ginkgo Biloba leaf extract that contains two major active constituents (24% ginkgo flavoglycosides and 6% terpene lactones), was purchased from Indena, Settala, Italy, and kindly provided by Yung Shin Pharmaceutical Ind. Co., LTD, Taiwan. The drug was dissolved in dimethyl sulfoxide (DMSO) to make a stock concentration. For experiments, the required concentrations of EGb were made by further dilution of the concentrated stock solution with culture medium. Polyclonal antisera against total extra-cellular signal regulated kinase (ERK)1, ERK2, p38, and JNK (for western blot) were obtained from Santa Cruz Biotechnology (Santa Cruz, CA). The antibodies recognizing c-Jun, c-Fos, ubiquitin, phosphorylated ERK, phosphorylated p38, and phosphorylated JNK were purchased from Cell Signaling Technology, Inc. (Beverly, MA). The anti-Flag and anti-HA antibodies were purchased from Sigma-Aldrich Chemical Company (St. Louis, MO) and GeneTex (Irvine, CA, USA), respectively. The proteosome inhibitor MG-132 (ZLeu-Leu-Leu-CHO) and the inhibitor of ubiquitin-activating enzyme E1, 4[4-(5-nitro-furan-2-ylmethylene)-3,5-dioxo-pyrazolidin-1-yl]-benzoic acid ethyl ester (PYR-41), were purchased from Calbiochem (Merck Biosciences, Darmstadt, Germany). ELISA kits for determination of chemokines, such as RNATES, MIP-1α, MIP-1β, and MCP-2 were purchased from R&D. Unless specified otherwise, the rest of the reagents were purchased from Sigma-Aldrich Chemical Company (St. Louis, MO).

### Preparation of chondrocytes

After obtaining written informed consent with prior approval of the Institutional Review Board of the Tri-Service General Hospital, the cartilage from OA patients who received total knee or total hip joint replacement was obtained aseptically. The preparation of chondrocytes from cartilage was performed as previously described [23]. In brief, full thickness articular cartilage was removed from the underlying bone and cut into pieces measuring 0.5 cm^2^. After enzymatic digestion with 2 mg/ml protease (Sigma) in serum-free Dulbecco's modified Eagle's medium (DMEM)/antibiotics (Invitrogen, Carlsbad, CA, USA) for 1 h at 37°C in an atmosphere of 5% CO_2_, the specimens were digested with 0.25 mg/ml collagenase I and 500 U/ml hyaluronidase in DMEM medium containing 10% fetal bovine serum overnight. After passing through a cell strainer, cells were collected and seeded at 6–8×10^6^ cells in T75 flasks in DMEM containing 10% FBS and antibiotics for 3 to 4 days before use. For some experiments, the chondrocyte cell line CHON-002 was used (American Type Culture Collection).

### Measurement of NO production

NO synthesis was determined by measuring its stable end product, nitrite, in supernatants as described previously [24]. The Griess reaction was performed with concentrations of nitrite measured by a spectrophotometer. In brief, an aliquot (100 µl) of culture supernatant was incubated with 50 µl of 0.1% sulfanilamide in 5% phosphoric acid and 50 µl of 0.1% *N*-1-naphthyl-ethylenediamine dihydrochloride. After 10 min of incubation, the absorbance was measured at a wavelength of 550 nm with a microplate reader (Tecan, Grodig, Australia).

### Reverse transcriptase-polymerase chain reaction (RT-PCR) and real time RT-PCR

RT-PCR was performed as previously reported [24]. In brief, total RNA was isolated after lysing cells with Trizol reagent (Invitrogen, Carlsbad, CA, USA). After reverse transcription of RNA to cDNA, samples were subjected to PCR. Consensus primers used in this study for iNOS were 5′-ACATTGATCAGAAGCTGTCCCAC-3′ and 5′-CAAAGGCTGTGAGTCCTGCAC-3′, which generated a 236-bp product. The primers used to amplify β-actin were 5′-CATGGTGGTGCCGCCAGACAG-3′ and 5′-ATGGCCACGGCTGCTTCCAGC-3′. Real-time quantification of c-Jun and GAPDH mRNA was performed according to the manufacturer's instructions (Power SYBR Green PCR Master Mix, Applied BioSystems, Foster City, CA). In brief, 5 ng of cDNA was amplified in a total volume of 20 µl consisting of 1x Master Mix and the gene-specific primers, which were added at a final concentration of 100 n*M*. The primer sequences were as follows: 5′-CCT TGA AAG CTC AGA ACT CGG AG-3′ and 5′-TGC TGC GTT AGC ATG AGT TGG C-3′ (for c-Jun); 5′-GTC ATC CAT GAC AAC TTC GG -3′ and 5′-GCC ACA GTT TCC CAG AGG -3′ (for GAPDH). The reactions were performed for 50 cycles with 95°C for denaturation and 60°C for annealing and extension on the ABI Prism 7000 Sequence Detection system (Applied BioSystems, Foster City, CA). The data were collected and the changes in gene expression following stimulation in the presence or absence of drugs were calculated.

### Nuclear extract preparation

Nuclear extracts were prepared as described previously [22]. Briefly, the cells (2×10^6^) were left at 4°C in 50 µl of buffer A [10 mM HEPES, pH 7.9, 10 mM KCl, 1.5 mM MgCl_2_, 1 mM dithiothreitol (DTT), 1 mM PMSF, and 3.3 µg/ml aprotinin] for 15 min with occasional gentle vortexing. The swollen cells were centrifuged at 25,000 g for 3 min. After removal of the supernatants, the pelleted nuclei were washed with 50 µl of buffer A, resuspended in 20 µl of buffer C (20 mM HEPES, pH 7.9, 420 mM NaCl, 1.5 mM MgCl_2_, 0.2 mM EDTA, 25% glycerol, 1 mM DTT, 0.5 mM PMSF, and 3.3 µg/ml aprotinin), and incubated at 4°C for 30 min with occasional vigorous vortexing. Then the mixtures were centrifuged at 25,000 g for 30 min and the supernatants were used as nuclear extracts.

### Electrophoretic mobility shift assay (EMSA)

The DNA-binding activity of NF-κB and AP-1 was determined by EMSA, using nuclear extracts prepared from chondrocytes as described previously [23]. The oligonucleotides containing NF-κB and AP-1 binding sites purchased from Promega were used as DNA probes. The probes were radio-labeled with [γ-^32^p]ATP using T4 kinase (Promega). For the binding reaction, the radio-labeled NF-κB or AP-1 probe was incubated with 5 µg of nuclear extract. The binding buffer contained 10 mM Tris-HCl (pH 7.5), 50 mM NaCl, 0.5 mM EDTA, 1 mM DTT, 1 mM MgCl_2_, 4% glycerol, and 2 µg poly(dI-dC). Binding reaction was carried out for 20 min at room temperature. If un-radiolabeled competitive oligonucleotides or monoclonal antibodies for supershift assays were added, they were preincubated with nuclear extracts for 30 min before adding radiolabeled probes.

### Western blotting

ECL western blotting (Amersham-Pharmacia, Piscataway, NJ) was performed as previously described [23]. Briefly, equal amounts of whole cellular extracts were analyzed on 10% SDS-PAGE and transferred to a nitrocellulose filter. For immunoblotting, the nitrocellulose filter was incubated with Tris-buffered saline containing 5% non-fat milk for 2 h and then blotted with antibodies against specific proteins for another 2 h at room temperature. After washing with milk buffer, the filter was incubated with rabbit anti-mouse IgG or goat anti-rabbit IgG conjugated to horseradish peroxidase at a concentration of 1∶5000 for 30 min. The filter was then incubated with the ECL substrate and exposed to X-ray film (Kodak).

### Chemotaxis analysis

The transwell migration assay was performed as previously described, with the exception that THP-1 cells were used instead of dendritic cells [25]. The cell culture supernatants were added in the lower chambers of a transwell plate with a pore size of 5 µM (Costar, Cambridge, MA) and pre-equilibrated to 37°C. Subsequently, THP-1 cells (1×10^6^/ml) were added in the upper chambers of the transwell plate. Migration was carried at 37°C in 5% CO_2_ for 4 h. Then cells migrating from the upper chamber to the lower chamber were counted by flow cytometry.

### Transfection assays

The transient transfection was performed by the calcium phosphate precipitation method. In brief, chondrocytes were transfected with the DNA-calcium phosphate preparation, which consisted of 5 µg/well of the reporter plasmid AP-1-firefly luciferase (Stratagene, La Jolla, CA, USA) and 1 µg/well of the internal control plasmid TK-Renilla luciferase (Promega, Madison, WI, USA) in 10% FBS culture medium. Approximately 4 h after transfection, the chondrocytes were washed and replaced with fresh serum-free medium in the presence or absence of EGb for 48 h and then stimulated or without IL-1 for another 24 h. The cell pellets were harvested and the total cell lysates were prepared for measuring luciferase activity using a luminometer according to manufacturer's instructions (Promega). The renilla luciferase values were used to normalize each sample for transfection efficiency. Results are expressed as fold induction of luciferase activity.

### Immunofluorescence examination

CHON-002 cells were seeded on Lab. Tek II chamber slides (Nalge Nunc International, IL, USA). After the indicated treatment and stimulation procedures, cells were washed with PBS and fixed in ice-cold methanol. Slides were treated with 5% FBS to block non-specific binding and then incubated with anti-c-Jun antibody followed by FITC-conjugated goat anti-rabbit antibody (BD Pharmingen, San Jose, California USA). The nucleus was visualized by incubating cells with 10 µg/ml of the fluorescent DNA binding dye 4, 6-diamidino-2-phenylindole (DAPI) at 37°C for 30 min. The expression of c-Jun or DAPI was visualized under a fluorescence microscope.

### Construction and generation of lentivirus carrying Flag(3x)-c-Jun and ubiquitin-hemagglutinin (HA)

cDNA of both c-Jun (BCRC # g1005035H03, Bioresource Collection and Research Center of Taiwan) and ubiquitin-HA (plasmid #17608, Addgene) were used to exogenously express Flag-c-Jun and ubiquitin-HA in chondrocytes. The construct of c-Jun was first cloned into the vector p3XFLAG-CMV-14 (Sigma) and then Flag-tagged-c-Jun and ubiquitin-HA were sub-cloned into the lentivector pLKO_AS3w.puro (National RNAi Core facility in Taiwan). The expression constructs were co-transfected with the packaging plasmid pCMV-ΔR8.91 and the envelop plasmid pMD.G to 293T cells to produce recombinant lentivirus. The virus-containing supernatant was harvested and the virus titer (relative infection unit/ml) was determined using a relative viral titrating method according to RNAi Core Facility. To generate Flag-c-Jun and ubiquitin-HA transduced cell lines, 50–70% confluent cell cultures were transduced with lentivirus carrying Flag-c-Jun and lentivirus containing ubiquitin-HA together at a multiplicity of infection (MOIs) of 2.5 in the presence of 8 µg/ml polybrene. Six days after transduction, cells were equally divided for further treatment and analysis.

### Ubiquitin Conjugation Assays

Treated cells were lysed in RIPA buffer. An equal amount of total cell lysates was pre-cleared and immunoprecipitated with Anti-Flag M2 antibody (SI-F1804 from Sigma) and protein A Sepharose and then mixed on a rotator overnight at 4°C. The beads were then washed 4 times with RIPA buffer. Purified proteins were eluted by boiling the beads in 2× sample loading buffer and analyzed by immunoblotting.

### Statistical Analysis

The results are expressed as means ± standard deviation (S.D.), as appropriate. One-way ANOVA analysis was used to determine significant differences. A P value <0.05 was considered to represent statistical significance.

## Results

### Effect of EGb on IL-1-stimulated chemokine production and cell migration

**Figure 1:**
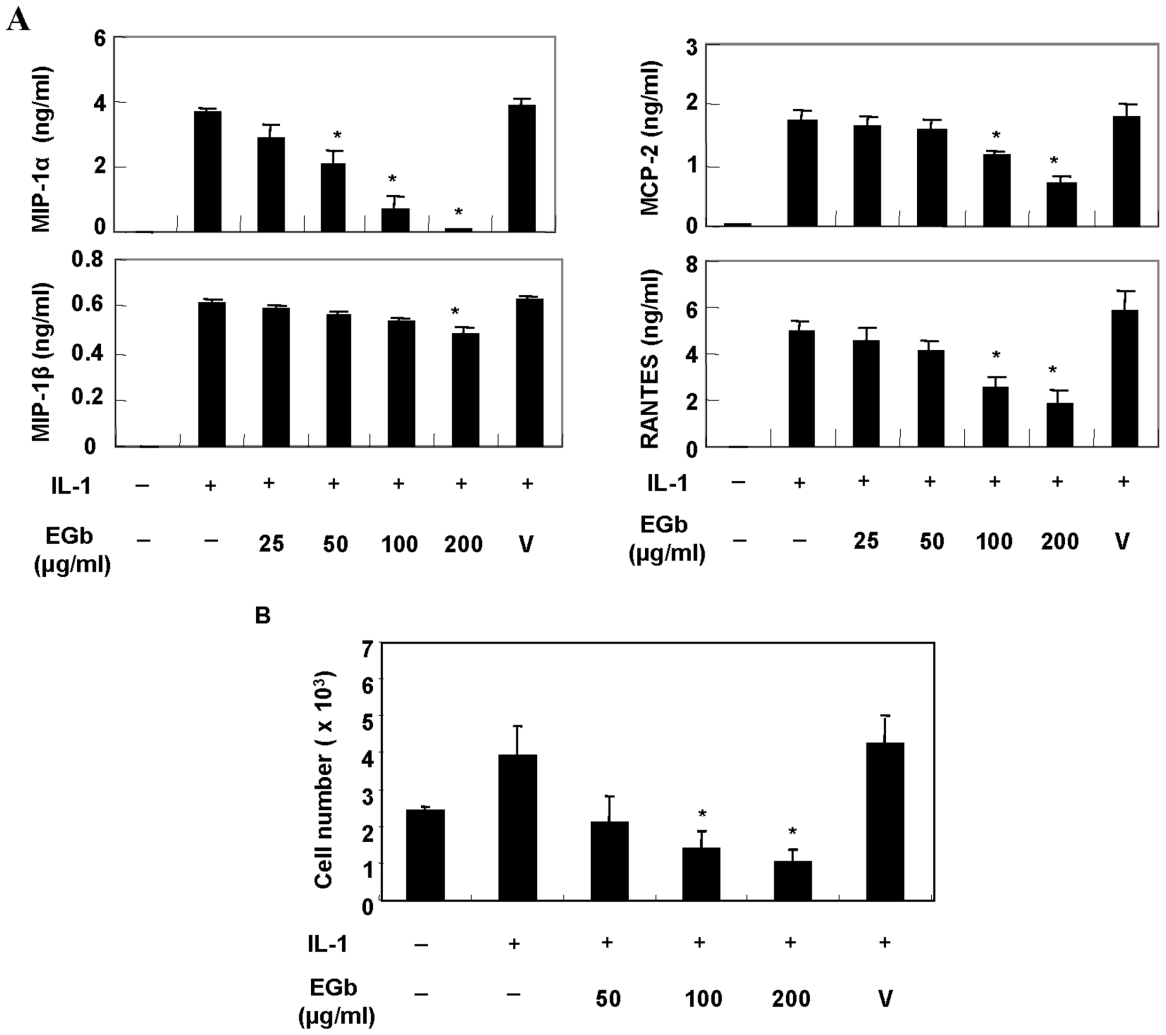
EGb treatment inhibited IL-1-induced chemokine production and cell migration. Chondrocytes, 3×10^5^ for each condition, were treated with various concentrations of EGb for 72 h and then stimulated with IL-1 (5 ng/ml) for another 24 h. The supernatants were collected and the concentrations of RANTES, MCP-2, MIP-1α and MIP-1β were measured by ELISA (A). Equal numbers of THP-1 cells were loaded in the upper chambers of the transwell cassette and the lower chambers were loaded with IL-1-treated cells that had been pretreated with or without EGb. Migration was carried out at 37°C in 5% CO_2_ for 4 h. Then cells migrating from the upper chambers to the lower chambers were counted by flow cytometry (B). *Denotes statistical significance (P<0.05) vs stimulated control cells. V: vehicle (DMSO in this study).

The results of the trypan blue exclusion assay and the 3-[4,-Dimethylthiazol-2-y]-2,5-diphenyl-tetrazolium bromide (MTT) colorimetric assay indicated that the EGb concentrations examined in this study were not cytotoxic (data not shown). We examined the effects of EGb on IL-1-induced chemokine production in human chondrocytes. Chondrocytes were pre-incubated in the presence or absence of various concentrations of EGb for 72 h and then stimulated with IL-1 (5 ng/ml). After 72 h, the supernatants were collected and chemokine concentrations were measured. EGb treatment resulted in a dose-dependent reduction in IL-1-induced RANTES, MCP-2, MIP-1α and MIP-1β expression (Fig. 1A). Cell migration assays were then performed to evaluate the significance of the EGb-induced reduction in chemokine expression. The results of the assays revealed that EGb-treated culture supernatants attracted fewer THP-1 cells than untreated culture supernatants (Fig. 1B).

### Effect of EGb on IL-1-induced NO production and iNOS expression

**Figure 2:**
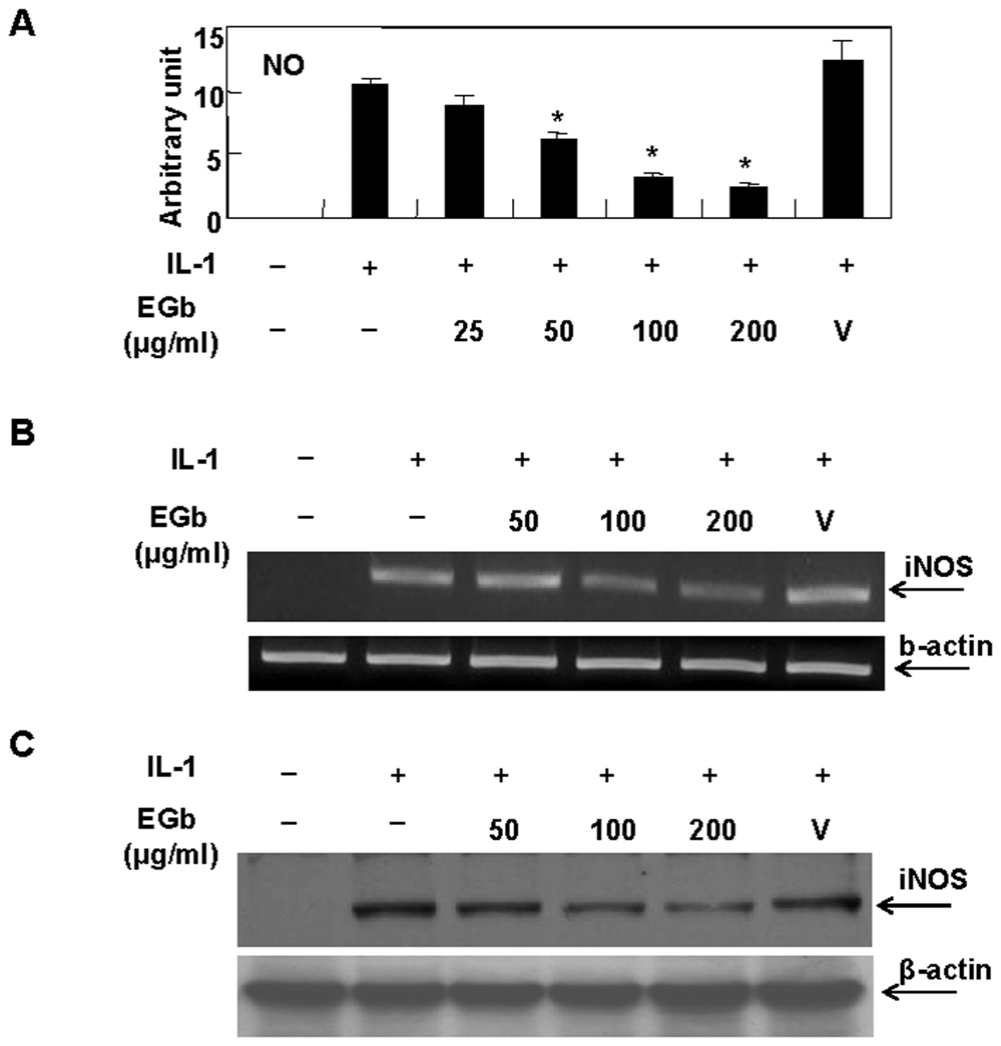
Inhibition of IL-1-induced NO production and iNOS expression by EGb. Chondrocytes, 1-2×10^6^ for each condition, were pretreated with various concentrations of EGb for 72 h and then stimulated with IL-1 for another 24 h. The culture supernatants were collected for NO measurement by Griess assays (A). The cells were collected for the determination of mRNA expression by RT-PCR (B) or protein expression by western blot (C). The representative results of at least three independent experiments using different donor cells are shown. V: vehicle.

We determined whether EGb had any effect on IL-1-induced NO production. Chondrocytes were pretreated with various concentrations of EGb for 72 h and then stimulated with IL-1 for 24 h. The levels of NO in culture supernatants, the amount of iNOS mRNA and the level iNOS protein expression in collected cells were then measured individually. Our results showed that EGb suppressed IL-1-induced NO production in a dose-dependent manner (Fig. 2A). EGb also inhibited IL-1-induced iNOS mRNA expression (Fig. 2B). By western blot analysis, we demonstrated that EGb reduced protein levels of iNOS induced by IL-1 stimulation (Fig. 2C).

### Effect of EGb on IL-1-induced AP-1 and NF-κB DNA-binding activity

**Figure 3:**
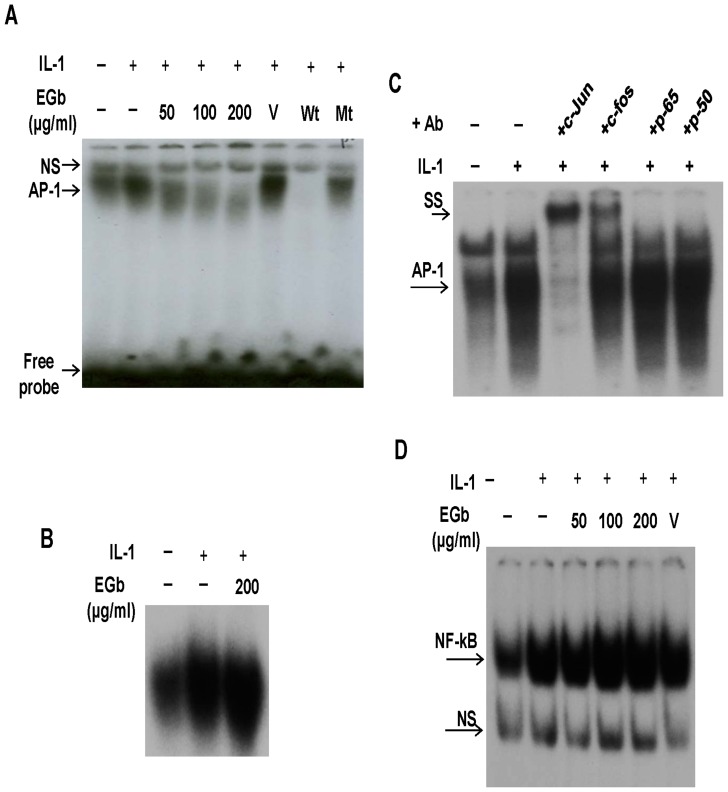
Suppression of IL-1-induced AP-1, but not NF-κB, DNA-binding activity by EGb. Chondrocytes, 1–2×10^6^ for each condition, were pretreated with or without various doses of EGb for 72 h and then stimulated with IL-1 for another 24 h. The cells were collected and the nuclear extracts were prepared for EMSA analysis. For competition studies, the competitors with unradiolabeled wild-type (Wt.) or mutant (Mt.) AP-1 oligonucleotides were added 30 min before addition of the radiolabeled AP-1 probe. The final reaction mixture was analyzed on a 6% native polyacrylamide gel (A). In (B), the IL-1-stimulated cellular nuclear extracts were incubated with or without 200 µg/ml EGb for 30 min before EMSA analysis. In (C), the experiments were performed similarly with (A), except that monoclonal antibodies against c-Jun, c-Fos, p65 or p50 were used instead of oligonucleotide competitors. EMSA analysis was also conducted to determine the NF-κB DNA-binding activity (D). NS, non-specific; SS, supershifts.

To evaluate the mechanisms responsible for the EGb-mediated effects, we examined the possible inhibitory effects of EGb on DNA-binding activity of AP-1 and NF-κB in IL-1-stimulated chondrocytes. There was strong AP-1 DNA-binding activity induced after IL-1 stimulation. In the presence of EGb, such activity was significantly suppressed (Fig. 3A). The AP-1 DNA-binding complex was specific because it could be competed only by wild-type but not by mutant unradiolabeled AP-1 oligonucleotides. To examine whether EGb directly suppressed AP-1 binding to its cognate oligonucleotides, we incubated nuclear extracts from IL-1-stimulated cells with EGb and then performed EMSA analysis. The result showed that EGb did not directly interfere with DNA binding of AP-1, indicating that EGb might affect AP-1 upstream signals (Fig. 3B). Results of antibody supershift assays demonstrated that the AP-1 DNA-binding complex contained c-Jun and c-Fos but not p65 or p50 (Fig. 3C). Although the NF-κB DNA-binding activity was also induced by IL-1, EGb had no effect on it (Fig. 3D).

### Effect of EGb on IL-1-induced MAP kinase activation

**Figure 4:**
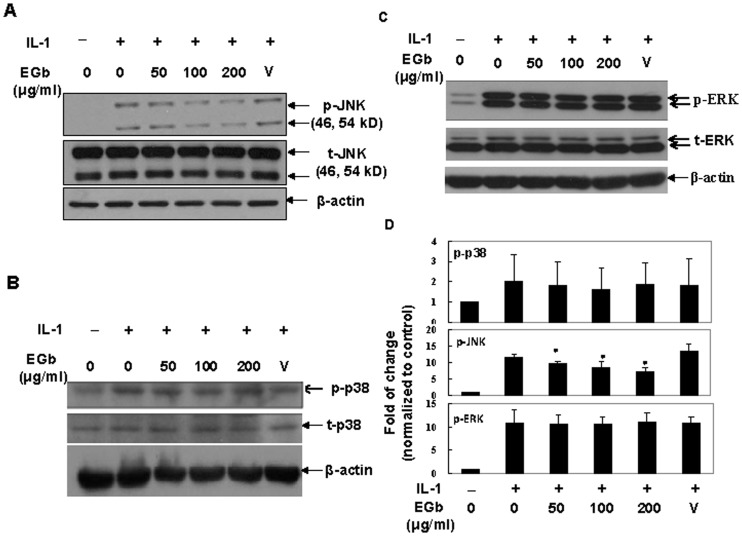
EGb inhibited IL-1-induced JNK but not p38 or ERK activity. Chondrocytes were pretreated with various concentrations of EGb for 72-1 for 15 min. The total cell lysates were analyzed by western blot using antibodies against un-phosphorylated or phosphorylated JNK (A), p38 (B) or ERK (C). The fold inductions of densitometric intensity of individual proteins equalized with β-actin, compared to those in un-stimulated samples, from more than 3 independent experiments using different donor cells (n = 5 for JNK and n = 3 for ERK and p38) are shown (Fig. 4D).

The activation of AP-1 relies on its upstream MAP kinases. Therefore, we determined which IL-1-induced MAP kinases were targeted by EGb. Total cell lysates from IL-1-treated cells that had been pretreated with or without EGb were analyzed by western blot using antibodies against phosphorylated forms of JNK, p38, and ERK. As shown in Fig. 4A, EGb treatment effectively reduced the expression of phosphorylated forms of JNK. In contrast, IL-1-induced phospho-p38 (Fig. 4B) and phospho-ERK (Fig. 4C) were not affected by EGb. The fold inductions of densitometric intensity of individual proteins equalized with β-actin, compared with those in un-stimulated samples are shown in Fig. 4D.

### Effect of EGb on c-Jun mRNA and protein levels

**Figure 5:**
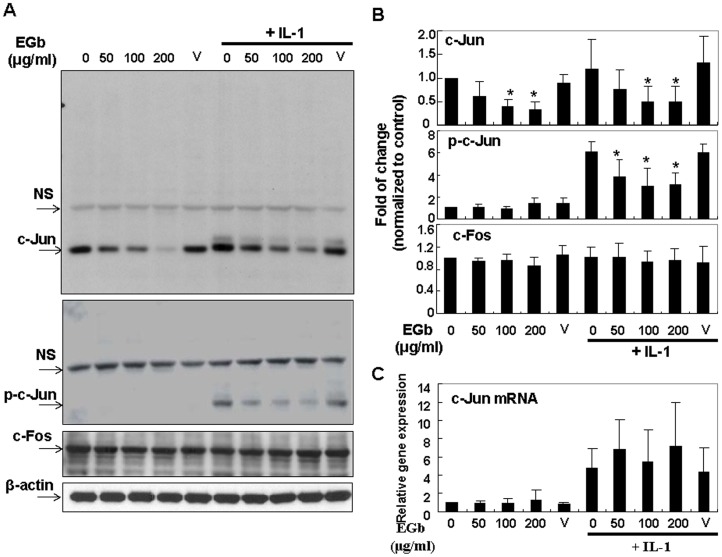
EGb induced c-Jun degradation. Chondrocytes were pretreated with or without various concentrations of EGb for 72-1 for another 15 min. The total cell lysates were prepared and the levels of c-Jun, phosphorylated c-Jun, c-Fos and β-actin were determined by western blot (A). In (B), the fold inductions of relative densitometric intensity of c-Jun, phosphorylated c-Jun and c-Fos, adjusted by β-actin intensity, from more than 3 independent experiments using different donor cells are shown. In (C), total RNA was prepared from the collected cells and RT-PCR was performed to measure c-Jun and GAPDH mRNA expression. The pooled results from 4 independent experiments examining different donor cells are shown.

We were surprised to find that the intensity of the AP-1 DNA-binding complex in cells treated with 100 µg/ml EGb was far lower than the basal level (Fig. 3A). This observation could not be explained by the incomplete inhibition of JNK activity by EGb (Fig. 4A). We, therefore, wondered whether EGb may transcriptionally or post-transcriptionally regulate the levels of c-Jun or c-Fos. Chondrocytes were treated with EGb in the presence or absence of IL-1 and the total cell lysates were used to determine c-Jun levels by western blot. The results indicated that EGb could effectively reduce the levels of c-Jun in a dose-dependent manner and that this effect was not related to the presence or absence of IL-1 (Fig. 5A, upper panel). In addition, EGb potently reduced the level of phosphorylated c-Jun (Fig. 5A, middle panel). Under the same condition, c-Fos was not affected by EGb. Figure 5B showed the fold inductions of relative densitometric intensity of c-Jun, phosphorylated c-Jun, and c-Fos, adjusted by β-actin intensity, from 3-5 independent experiments using different donor cells. The results of real time (RT)-PCR analysis showed that EGb did not affect IL-1-induced c-Jun mRNA expression (Fig. 5C). The results, therefore, suggest that EGb regulates c-Jun expression, at least in part, through post-transcriptional mechanisms.

### Effect of EGb on c-Jun degradation in CHON-002 cells

**Figure 6:**
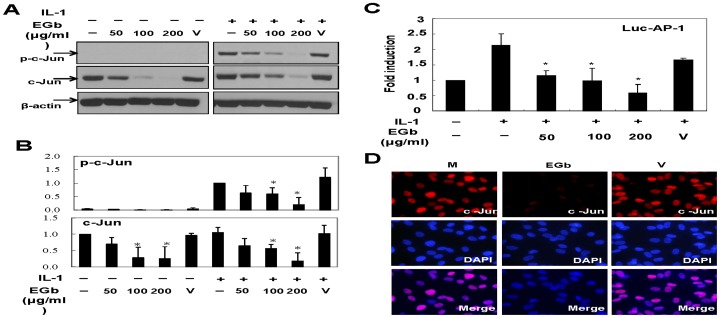
Effect of EGb on c-Jun expression in a human chondrocyte cell line CHON-002. CHON-002 cells were pretreated with various doses of EGb for 72 h and then stimulated with or without IL-1 for another 15 min. The total cell lysates were prepared and the expression of phosphorylated c-Jun, total c-Jun, and β-actin was determined by western blot (A). The results from three independent experiments are shown in (B). In (C), chondrocytes were transfected with DNA-calcium phosphate preparation containing 5 µg/well reporter plasmid AP-1-firefly luciferase and 1 µg/well internal control plasmid TK-Renilla luciferase. Four hours after transfection, the cells were washed and treated with different concentrations of EGb for 48 h followed by stimulation with IL-1 for another 24 h. The total cell lysates were prepared and the luciferase activity was determined. In (D), CHON-002 cells were treated with or without 200 µg/ml EGb for 72 h and the intensities of c-Jun and DAPI were determined by immunofluorescence staining as described in the Materials and Methods section.

We then examined the effects of EGb on CHON-002 cells, a human chondrocyte cell line that contains abundant c-Jun protein in resting status. Similar to the observations in primary chondrocytes, EGb potently reduced both basal and IL-1-induced c-Jun levels (Fig. 6A and 6B). Transfection assays revealed that IL-1 stimulation resulted in a 2.2-fold increase in AP-1 luciferase reporter activity and that EGb significantly inhibited such an effect (Fig. 6C). Furthermore, the levels of c-Jun protein were verified by immunofluorescence staining and directly visualized by a fluorescence microscope. The results shown in Fig. 6D indicated that after incubation with EGb, c-Jun fluorescence intensity decreased. These results suggest that EGb directly affects c-Jun homeostasis.

### EGb-mediated c-Jun degradation could be blocked by a proteosome inhibitor MG-132 in both CHON-002 cells and human chondrocytes

**Figure 7:**
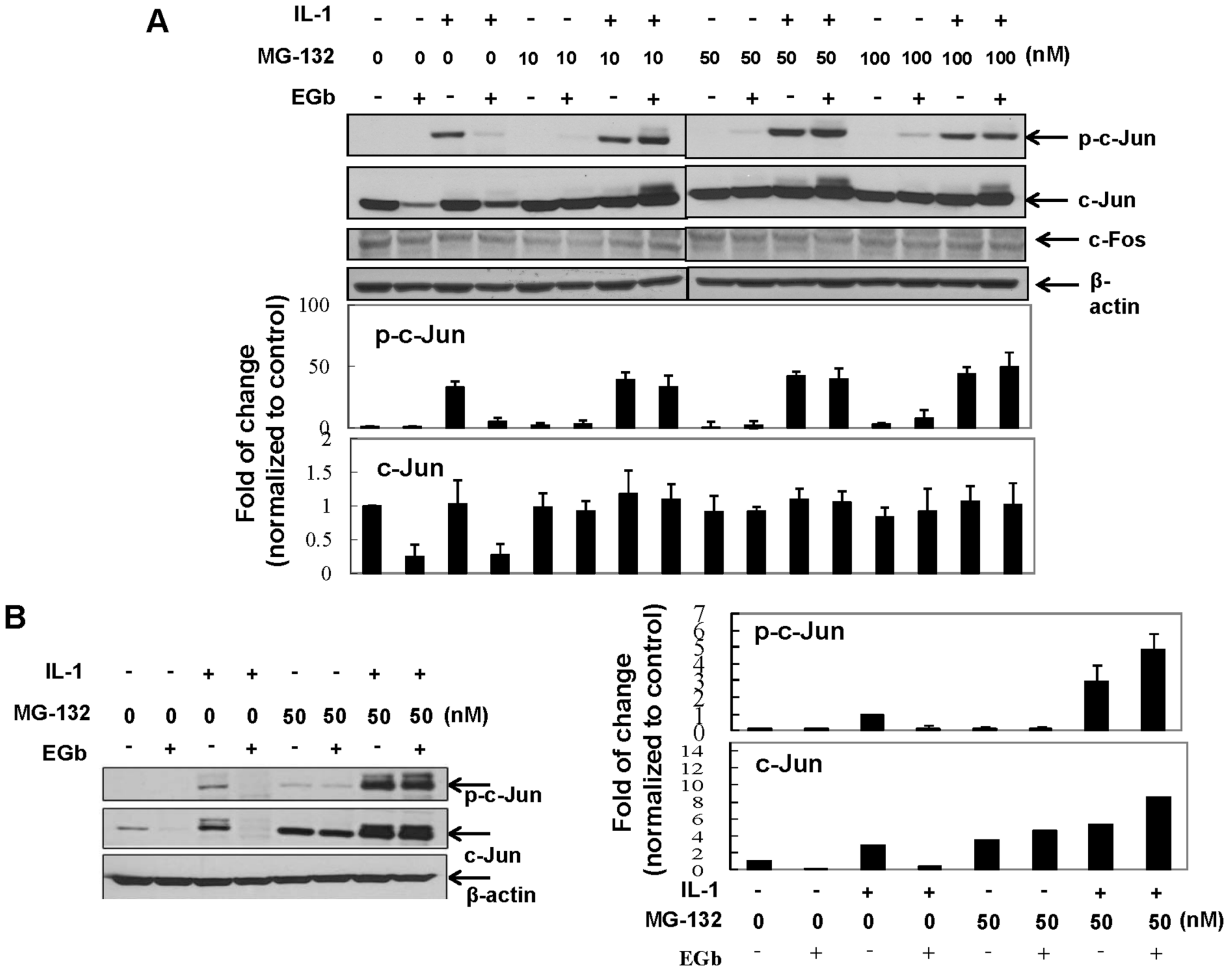
EGb caused c-Jun degradation via the proteosome pathway. CHON-002 cells were pretreated with 200 µg/ml EGb in the presence or absence of various doses of MG-132 for 72 h and then stimulated with or without IL-1 (5 ng/ml) for another 15 min. The total cell lysates were prepared and the expressions of phosphorylated c-Jun, total c-Jun and β-actin were determined by western blot (A). Similarly, the effects of the proteosome inhibitor MG-132 on EGb-induced degradation of c-Jun were examined in primary human chondrocytes (B). The representative results from 3 independent experiments are shown.

The mechanisms responsible for EGb-mediated degradation of c-Jun were further examined. CHON-002 cells were pre-treated with EGb for 72 h in the presence or absence of various doses of the proteosome inhibitor MG-132 and then treated with or without IL-1 for another 15 min. Whole cell lysates were collected for the determination of c-Jun, phosphorylated c-Jun, or β-actin level by western blotting. As shown in Fig. 7A, treatment with MG-132 at various doses (10–100 nM) significantly prevented EGb-mediated degradation of c-Jun. Similar observations were demonstrated in primary chondrocytes (Fig. 7B, left and right panels).

### EGb treatment resulted in c-Jun degradation in a ubiquitination-dependent manner

**Figure 8:**
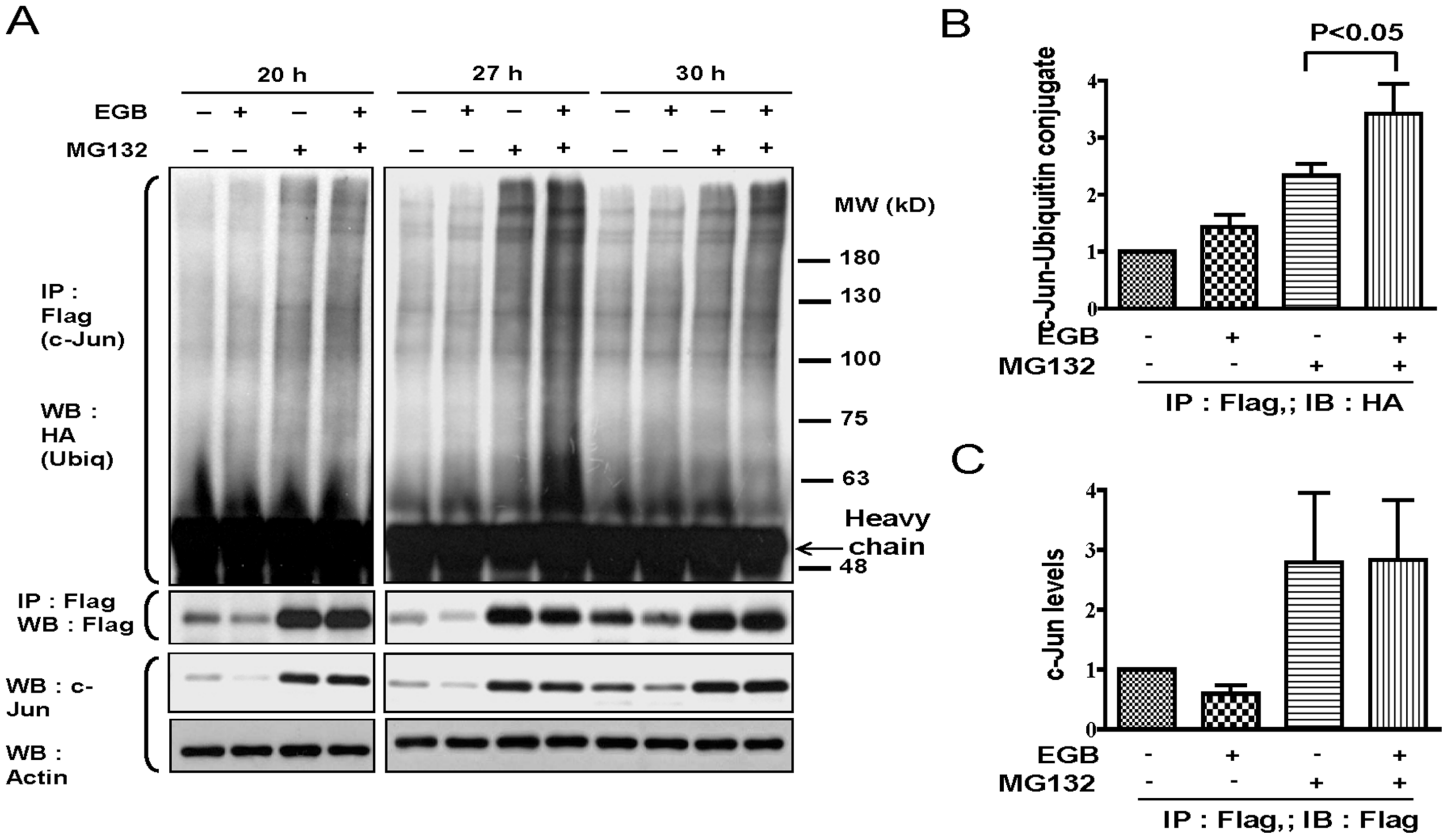
Involvement of ubiquitination-dependent process in EGb-mediated c-Jun degradation. CHON-002 cells were transfected by lentiviruses carrying Flag-c-Jun or HA-ubiquitin gene. Six days after transfection, the cells were treated with or without EGb for various time points and the total cell lysates were immunoprecipitated with antibodies against FLAG and then western blotted with antibodies against HA (A). Meanwhile, the total lysates were immunoblotted with antibodies against c-Jun or β-actin (A). The fold inductions of levels of c-Jun-ubiquitin conjugates at various time points in which the conjugate in un-treated cell lysates was taken as one fold are shown (B). Similar to (B), the levels of total c-Jun in different conditions are shown (C). The representative results from at least 3 independent experiments are shown.

**Figure 9:**
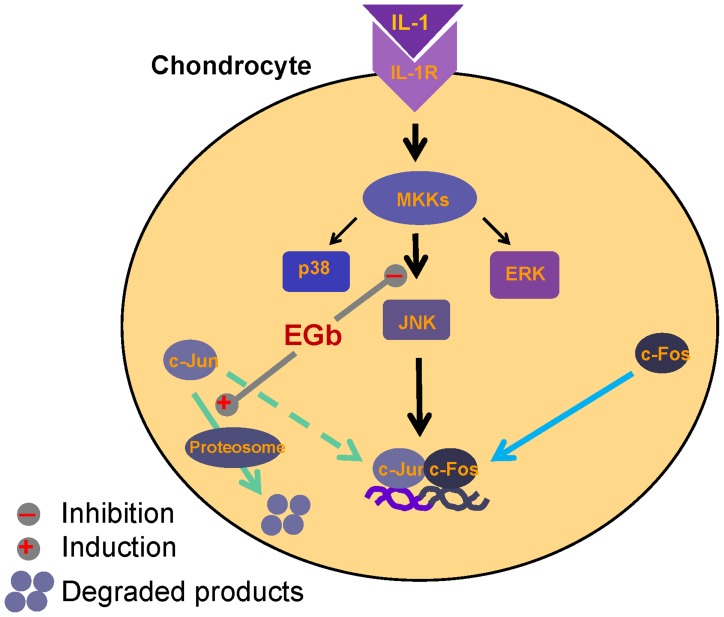
EGb independently and individually blocked IL-1-mediated activation of JNK and induced degradation of c-Jun in chondrocytes. Binding of IL-1 to its receptor causes activation of MAPK upstream kinases (MKKs) and subsequently MAPKs, including JNK, p38 and ERK. Activated MAPKs then stimulate AP-1. EGb through inhibiting JNK activity and inducing ubiquitination-dependent degradation of c-Jun suppressed IL-1-mediated effects in chondrocytes.

Both ubiquitination-dependent and ubiquitination-independent mechanisms have been observed in proteosome-mediated degradation processes in various proteins. We were interested to know which mechanism is involved in EGb-mediated c-Jun degradation. To elucidate the process, lentivirus carrying a Flag-tagged c-Jun or HA-tagged ubiquitin gene was transfected into CHON-002 cells as described in the Materials and Methods. Six days after transfection, cells were equally divided and treated with 200 µg/ml EGb for various time points. One group of cells was also treated with 0.5 µM MG-132 2 h before cell collection. We found that the level of ubiquitinated c-Jun was markedly higher in cells that had been exposed to MG-132 but that EGb treatment alone also marginally increased the level of ubiquitinated c-Jun (Fig. 8A and 8B). Furthermore, the expression level of c-Jun-ubiquitin conjugate was significantly higher in cells that had been treated with EGb and MG-132 than in cells that had been treated with MG-132 alone (Fig. 8B). The levels of total c-Jun appeared to be similar in both EGb+MG-132 and MG-132 treatment groups (Fig. 8C). To more specifically address this issue, CHON-002 cells were pre-treated with or without EGb for 19 h and then treated with 25 µM PYR-41, a specific E1-type ubiquitin-activating enzyme inhibitor, for 8 h. The total cell lysates were collected and the expression of c-Jun was determined by western blotting. The results demonstrated that PYR-41 treatment successfully prevented EGb-mediated c-Jun degradation (data not shown). Collectively, the results suggest that EGb-mediated c-Jun degradation likely involves the ubiquitination process (summarized in Fig. 9).

## Discussion

Pain killers are the only effective therapeutic regimen for patients with OA; however, long-term use of many analgesic agents can result in adverse effects on the gastrointestinal and cardiovascular systems. Therefore, components from food or drink or even dietary supplements that maintain modest immunomodulatory properties, such as resveratrol [26], may be reasonable alternatives to achieve symptomatic improvement in patients with OA.

In a double-blind, placebo-controlled clinical trial, we found that EGb administration resulted in improved joint pain symptom scores in patients with OA (manuscript in preparation). This study was, therefore, undertaken to examine whether EGb inhibits the inflammatory response in chondrocytes. We found that EGb has immunomodulatory effects and that these effects are governed by its ability to downregulate IL-1-induced chemokine production and iNOS-NO activation, and by its ability to indirectly suppress inflammatory cell migration. To our surprise, we also found that EGb inhibited IL-1-stimulated JNK activity and induced ubiquitination-dependent degradation of c-Jun in chondrocytes (summarized in Fig. 9). To the best of our knowledge, this duel immunomodulatory mechanism of EGb has not been reported previously.

The iNOS-NO signaling pathway has been the subject of many studies on the pathogenesis of OA. In experimental OA models, the inhibition of NO production significantly reduces progression of cartilage damage [1]. In addition, it has been shown that the suppression of the iNOS-NO pathway prevents chondrocytes from undergoing apoptosis [27]. In this regard, the dose-dependent inhibition of IL-1-induced iNOS-NO activation by EGb should bring predictable benefits to patients with OA. In addition to its role in the pathogenesis of OA, the iNOS-NO pathway is also crucial in cerebrovascular dysfunction and peripheral vascular disorders [28]. There is evidence that EGb treatment results in a reduction of oxidized low-density lipoprotein-induced functional damage in endothelial cells [29]. In addition, in our previous study, we found that EGb inhibits the AP-1 signaling pathway in T cells [22]. Collectively, the accumulated evidence indicates that EGb is a potential therapeutic agent for patients with T cell-mediated pathologies as well as for patients with atherosclerosis, cardiovascular disease, or cerebrovascular disease

In this study, we also investigated the effect of EGb on chemokines. We found that EGb suppressed the production of RANTES, MCP-2, MIP-1α and MIP-1β and hence a reduction in the number of THP-1 cells migrating toward EGb-treated cell culture medium. Such effects would greatly reduce the vicious circle triggered by chemokines in inflammatory reactions. The results from other studies have shown that suppression of AP-1 activation alone is sufficient to inhibit chemokine production [12], [30].

The inflammatory process in patients with OA involves activation of the JNK-AP-1 signaling pathway, a phenomenon demonstrated in several OA models. Mechanical stress is an important factor in the pathogenesis of OA. The results also indicate that *ex vivo* cartilage compression can stimulate phosphorylation of JNK [31]. In addition, fluid shear stress, another OA-inducing mechanism, has also been shown to activate the JNK signaling pathway and to elicit an inflammatory response by up-regulating cyclooxygenase-2 [32]. In thermal stress-induced activation of chondrocytes, the suppression of JNK activity helps to maintain capacity of chondrocytes for proteoglycan synthesis [33]. Another example is in mechanical load-triggered differentiation in subchondral osteoblasts where the JNK-c-Jun/c-Fos signaling pathway regulates the critical process of the event [34]. By regulating the JNK signaling pathway, prostaglandin E_2_ has been shown to protect chondrocytes from IL-1-induced damage through increased expression of matrix metalloproteinase-1 and matrix metalloproteinase-13 [35]. Furthermore, studies have shown that JNK inhibitors are potential therapeutic agents for the management of a variety of inflammatory disorders [36], [37]. Accordingly, the inhibition of JNK activation by EGb suggests its potential in controlling several aspects of OA pathogenesis.

Our observation that EGb induced c-Jun degradation in a ubiquitination-dependent manner was unexpected. As a highly unstable oncoprotein, N-terminus phosphorylation of c-Jun by JNK and likely other protein kinases can stabilize and decrease c-Jun ubiquitination and degradation [38]. However, there are reports showing that JNK-mediated phosphorylation may accelerate c-Jun degradation by allowing its recognition by the E3 ligase complex [39]. Alternatively, JNK can phosphorylate E3 ligase Itch to regulate c-Jun degradation [40]. Our findings indicate that JNK might not be involved in EGb-mediated c-Jun degradation, given that JNK was not activated in resting chondrocytes. Nevertheless, the presence of ubiquitinated c-Jun and both MG-132- and PYR-41-suppressible c-Jun degradation confirms that the degradation process of c-Jun is mediated by the classical pathway. The results in Fig. 7 indicate that MG-132 by itself can increase c-Jun and phosphorylated c-Jun levels. These findings are consistent with observations made in rat mesangial cells [41]. According to Joshi-Barve et al [42], treatment of HepG2 cells with MG-132 induced AP-1 DNA-binding activity as well as stimulated phosphorylation of JNK. Deficiency of c-Jun results in embryonic lethality in mice. Therefore, the detailed function of c-Jun has not been fully addressed in many organ systems, including chondrocytes, in murine models. Currently, we are investigating several possible mechanisms governing EGb-induced c-Jun ubiquitination and degradation.

In conclusion, we have demonstrated that EGb has chondro-protective effects, namely through inhibiting IL-1-stimulated JNK activation and inducing ubiquitination-dependent c-Jun degradation in chondrocytes. The results indicate that EGb should be considered as an alternative therapeutic agent for the management of patients with OA.
